# Relación entre el estado nutricional y el índice triglicéridos/c-HDL en adolescentes atendidos en un hospital público

**DOI:** 10.47487/apcyccv.v1i4.83

**Published:** 2020-12-31

**Authors:** Edwar Paul Cachay-Barboza

**Affiliations:** 1 Nutricionista. Maestrando en Nutrición Clínica, Unidad de Posgrado Facultad de Medicina, Universidad Nacional Mayor de San Marcos, Lima, Perú. Universidad Nacional Mayor de San Marcos Unidad de Posgrado Facultad de Medicina Universidad Nacional Mayor de San Marcos Lima Peru; a Instituto Nacional Cardiovascular - INCOR, EsSalud, Lima, Perú Instituto Nacional Cardiovascular - INCOR, EsSalud Lima Perú

**Keywords:** Síndrome Metabólico, Enfermedades Cardiovasculares, Estado Nutricional, Adolescentes, Metabolic Syndrome, Cardiovascular Diseases, Nutritional Status, Adolescent

## Abstract

**Objetivo.:**

Determinar la relación del estado nutricional según el índice de masa corporal (IMC) y el índice triglicéridos/c-HDL en adolescentes atendidos en un hospital público.

**Materiales y métodos.:**

Estudio observacional, transversal y retrospectivo de la base de datos del programa de educación nutricional del Hospital I - Rioja - EsSalud, durante marzo de 2017 a junio de 2018. Se determinó el estado nutricional según el índice de masa corporal para la edad (IMC/edad) y el índice de Tg/c-HDL como la razón entre triglicéridos y el colesterol HDL.

**Resultados.:**

Estudio conformado por 130 adolescentes (70 mujeres y 60 varones) de entre 10 - 18 años de edad, quienes fueron atendidos en el servicio de nutrición del Hospital I - Rioja de EsSalud. La edad promedio de los adolescentes fue de 12,6 ± 2,2 años. Se obtuvo una media del índice de Tg/c-HDL de 2,41 ± 0,48; fue mayor en los varones (2,46 ± 0,51) que en las mujeres (2,37 ± 0,44); así mismo, la obesidad (2,70 ± 0,49), el sobrepeso (2,46 ± 0,41) y riesgo de bajo peso (2,36 ± 0,55) fueron los estados nutricionales que presentaron un índice Tg/c-HDL más elevado. Se encontró una correlación positiva y significativa (r=0,447, p=0,000) entre el IMC y el índice Tg/c-HDL.

**Conclusiones.:**

Encontramos una relación positiva y estadísticamente significativa entre el índice de masa corporal y el índice de Tg/c-HDL en esta población de adolescentes. Este índice fue mayor en el estado nutricional de obesidad y sobrepeso superando al valor de la media poblacional.

## Abstract (en)

**Objective.:**

To determine the relationship between nutritional status according to body mass index (BMI) and triglyceride index/c-HDL in adolescents treated in a public hospital.

**Materials and methods.:**

Observational, transversal, and retrospective study of the nutritional education database program of Hospital I - Rioja - EsSalud, from March 2017 to June 2018. The nutritional status was determined according to the body mass index for age (BMI/age) and the index of Tg/c-HDL as the ratio between triglycerides and HDL cholesterol.

**Results.:**

The study was made up of 130 adolescents (70 females and 60 males) between 10 - 18 years old, who were treated in the nutrition service of Hospital I - Rioja- EsSalud. The average age of the adolescents was 12.6 ± 2.2 years. The average Tg/c-HDL index was 2.41 ± 0.48; it was higher in males (2.46 ± 0.51) than in females (2.37 ± 0.44); likewise, obesity (2.70 ± 0.49), overweight (2.46 ± 0.41) and the risk of underweight (2.36 ± 0.55) were the nutritional states that presented the highest Tg/c-HDL index. A positive and significant correlation (r=0.447, p=0.000) was found between BMI and Tg/c-HDL index.

**Conclusions.:**

There was a positive and statistically significant relationship between the body mass index and the Tg / HDL-c index in this adolescent population. The Tg / HDL-c index was higher in the nutritional status of obesity and overweight, exceeding the value of the media population.

En la actualidad el número de personas con obesidad duplica a las de bajo peso ^1^. El exceso de peso (sobrepeso y obesidad) es considerado como una enfermedad crónica que genera trastornos fisiológicos, metabólicos y/o endocrinos, con cuadros de hipertrigliceridemia, hipercolesterolemia, resistencia a la insulina (RI), alteración de lipoproteínas, entre otras ^2^^,^^3^. Se estima que cada año fallecen aproximadamente 2,8 y 20 millones de personas a causa del exceso de peso y de enfermedades cardiovasculares (ECV) respectivamente, siendo esta la primera causa de muerte a nivel mundial ^4^^-^^7^. En el Perú, el exceso de peso supera el 32% en niños y afecta al 23% de los adolescentes ^8^^,^^9^, y es la selva la región con mayor prevalencia de exceso de peso (53,9%) en mayores de 15 años ^10^. Los factores que promueven activamente el desarrollo del exceso de peso aparecen desde la infancia o la adolescencia ^11^, en esta etapa es mayor el consumo de alimentos procesados y ultraprocesados con alto contenido en nutrientes como azúcar, sodio, grasas saturadas y trans ^12^^,^^13^.

El síndrome metabólico (SM) y el exceso de peso en la niñez y adolescencia estan relacionados con RI, hiperinsulinemia, alteración de lipoproteínas, aumento de citoquinas inflamatorias, y alteración funcional y estructural del endotelio vascular, lo que resulta en el desarrollo de ECV en la etapa adulta ^6^^,^^14^^,^^15^. La identificación temprana de los factores de riesgo para ECV en niños y adolescentes debe ser considerada como el eje principal en el diagnóstico precoz, para adoptar planes y estrategias en el cuidado y promoción de la salud; estos factores de riesgo son prevenibles y modificables, sobre todo en la etapa de la niñez y adolescencia ^16^. Se han recomendado diferentes indicadores y herramientas que ayudarían a identiﬁcar los factores de riesgo cardiovascular, entre ellas las medidas antropométricas ^17^, los análisis bioquímicos, los *screening* nutricionales, los índices clínicos, entre otros.

Se ha planteado el uso del valor de las lipoproteínas, y los índices generados entre cada una de ellas, como una medida más exacta para evaluar el riesgo cardiovascular y, sobre todo, la RI, debido a que las lipoproteínas son fácilmente determinadas en una muestra de perfil lipídico ^6^^,^^18^^-^^20^. En adultos, el índice de triglicéridos/colesterol-HDL (Tg/c-HDL) ha demostrado ser una herramienta predictiva de RI, SM, daño del endotelio vascular y de factores de riesgo cardiometabólico ^21^^,^^22^. En población infantil y adolescente este ratio se ha usado para identificar dislipidemias, hipertensión arterial (HTA) e incluso el SM ^23^^,^^24^.

Detectar los factores de riesgo cardiovascular en la infancia y la adolescencia puede llevarnos a conocer cuáles podrían ser sus consecuencias en la edad adulta; pero, muchas veces, estos factores de riesgo son difíciles de detectar o no se expresan en poblaciones pediátricas. En ese contexto es importante contar con herramientas prácticas, sencillas y eficaces como el índice triglicérido/c-HDL y el índice de masa corporal (IMC) como predictores de RI, SM y ECV en población pediátrica. Es por ello que el objetivo del presente estudio fue determinar la relación del estado nutricional según el índice de masa corporal y el índice triglicéridos/c-HDL en adolescentes atendidos en un hospital público.

## Materiales y métodos

Estudio descriptivo, transversal y retrospectivo ^25^ de la base de datos del programa de educación nutricional del Hospital I - Rioja de EsSalud durante marzo de 2017 a junio de 2018 utilizado para el monitoreo epidemiológico nutricional. Se consideró a la totalidad de los adolescentes de 10 - 18 años de edad de ambos sexos atendidos y registrados en la base de datos de dicho programa. Se obtuvo autorización escrita para el uso de la información del programa manteniéndose la protección de identidad e integridad de las personas.

Como variables de estudio se determinó y calculó el estado nutricional (EN) y el índice triglicéridos/c-HDL (Tg/c-HDL). El EN representó la situación nutricional actual de la persona resultado del análisis y de la comparación del indicador antropométrico IMC/edad, con valores de referencias internacionales establecidas por la Organización Mundial de la Salud (OMS), correspondiente a su edad y sexo. Se consideró como bajo peso, riesgo de bajo peso, normal, sobrepeso y obesidad cuando el indicador es menor a -2 desviaciones estándar (DS), -1 DS a -2 DS, 1DS a -1 DS y mayor a 2 DS respectivamente ^26^. El índice Tg/c-HDL es un valor matemático resultado de la división de la concentración de los triglicéridos entre el colesterol HDL, siendo utilizado como predictor de resistencia a la insulina, del desarrollo de síndrome metabólico y de la enfermedad cardiovascular. Se considera como óptimo, moderado y elevado cuando el índice es menor a 2, 2 a 3,8 y mayor a 3,8, respectivamente ^27^.

Para el análisis se usó el programa de SPSS en la versión 21 y Excel versión 2019; las variables numéricas se presentan como media ± desviación estándar (DS) o mediana con rango intercuartil, dependiendo de su distribución normal. Se usó la prueba t de Student para determinar la diferencia entre ambos sexos, y se calcularon los percentiles correspondientes al 25, 50, 75 y 95 para el índice triglicéridos/c-HDL. Se determinó la correlación de Pearson para las variables con distribución normal y se consideró como diferencia significativa un valor de p<0,05.

## Resultados

Se evaluaron 130 adolescentes (70 mujeres y 60 varones), las características de la población estudiada según el sexo se presentan en la Tabla 1**.** El índice Tg/c-HDL fue ligeramente superior en varones que en mujeres **(**Tabla 2**)**; sin embargo, esta diferencia no fue estadísticamente significativa (p= 0,322).

**Tabla 1:** Características de la población de estudio según sexo

Características	Varones (n=60)	Mujeres (n=70)	Total (n=130)	P^*^
	Media ± DS	Media ± DS	Media ± DS	
Edad (años)	12,8 ± 2,3	12,5 ± 2,1	12,6 ± 2,2	0,58
Peso (kg)	49,7 ± 14,6	48,1 ± 12,9	48,9 ± 13,6	0,52
Talla (cm)	150,3 ± 12,4	147,5 ± 8,6	148,8 ± 10,5	0,14
Triglicéridos (mg/dL)	115,9 ± 16,7	118,2 ± 15,1	117,1 ± 15,8	0,39
c-HDL (mg/dL)	48 ± 6,1	50,6 ± 6,1	49,4 ± 6,2	0,16
IMC (kg/m^2^)	21,6 ± 4,2	21,9 ± 4,5	21,8 ± 4,3	0,76

*p entre las medias ± DS (desviación estándar) según sexo; c-HDL (lipoproteína de alta densidad); IMC (índice de masa corporal).

**Tabla 2:** Índice Tg/c-HDL según sexo

Sexo	Tg/c-HDL Media ± DS	Pc25	Pc50	Pc75	Pc95
Varones (n=60)	2,46 ± 0,51	2,07	2,45	2,68	3,40
Mujeres (n=70)	2,37 ± 0,44	2,05	2,36	2,65	3,03
Total (n=130)	2,41 ± 0,48	2,05	2,42	2,66	3,28

p-valor = 0,322; Tg/ c-HDL (triglicéridos/lipoproteína de alta densidad); DS (desviación estándar); Pc (percentil).

**Figura 1:**
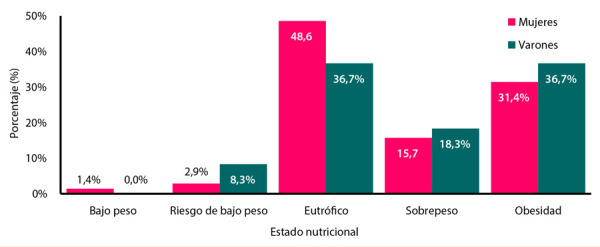
Estado nutricional según sexo.

Más del 50% de la población presentaba exceso de peso, de estos, más del 66% obesidad. Al segmentarse según sexo, 55% de varones (18,3% en sobrepeso y 36,7% en obesidad) y 47,1% de mujeres (15,7% en sobrepeso y 31,4% en obesidad) tuvieron exceso de peso **(**Figura 1**)**. Cruzando los resultados del índice Tg/c-HDL con el estado nutricional **(**Tabla 3**)**, se encontró que el índice Tg/c-HDL en varones fue mayor en el grupo que presentaba un estado nutricional de obesidad; mientras que en el grupo de las mujeres el mayor índice Tg/c-HDL estuvo en las personas con riesgo de bajo peso seguido de las mujeres con estado nutricional de obesidad.

**Tabla 3:** Índice Tg/c-HDL según el estado nutricional

Estado nutricional según IMC		Tg/c-HDL Media ± DS		
Clasificación nutricional	N (%)	Varones (n=60)	Mujeres (n=70)	Total (n=130)
Bajo peso	1 (0,8%)	(n=0) 0,00 ± 0,0	(n=1) 2,07 ± 0,0	2,07 ± 0,0
Riesgo bajo peso	7 (5,4%)	(n=5) 2,19 ± 0,48	(n=2) 2,79 ± 0,41	2,36 ± 0,55
Eutrófico	56 (43,1%)	(n=22) 2,24 ± 0,42	(n=34) 2,14 ± 0,44	2,18 ± 0,34
Sobrepeso	22 (16,9%)	(n=11) 2,50 ± 0,41	(n=11) 2,43 ± 0,36	2,46 ± 0,41
Obesidad	44 (33,8%)	(n=22) 2,72 ± 0,49	(n=22) 2,69 ± 0,56	2,70 ± 0,49
Total (n=130)	130 (100%)	2,46 ± 0,51	2,37 ± 0,44	2,41 ± 0,48

IMC (índice de masa corporal); DS (desviación estándar); Tg/ c-HDL (triglicéridos/lipoproteína de alta densidad).

**Figura 2:**
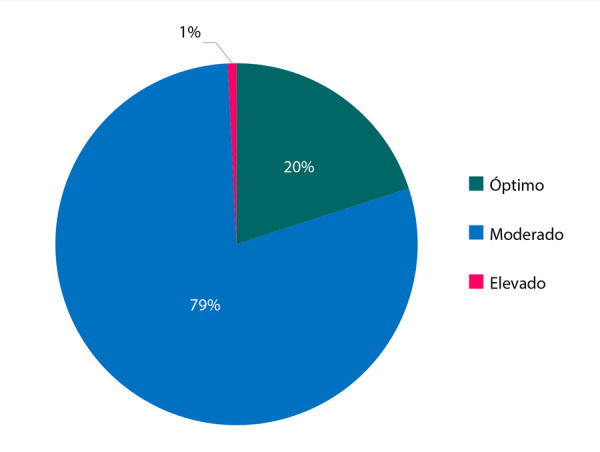
Estado del índice Tg/c-HDL.

**Figura 3:**
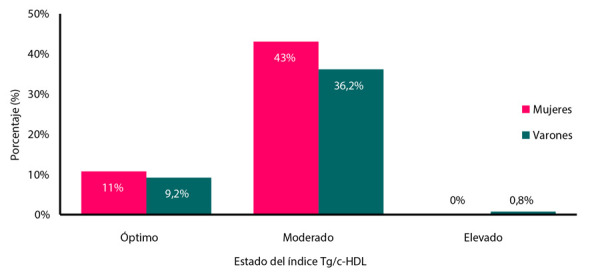
Estado del índice Tg/c-HDL según sexo.

Solo el 20% (n=26) de los adolescentes evaluados se encontraban con un índice Tg/c-HDL menor a 2,0 considerado como normal **(**Figura 2**)**. Las mujeres presentaron mejor índice Tg/c-HDL a comparación de los varones **(**Figura 3**)**.

Al valorar la correlación de Pearson entre el índice de masa corporal (IMC) con el índice Tg/c-HDL se encontró una correlación positiva moderada (r=0,447) con un p=0,000.

## Discusión

El índice de Tg/c-HDL encontrado en el presente estudio fue menor que en otras poblaciones; tal es así que en una población de la ciudad de Rosario - Argentina obtienen una media de 2,8 ± 0,39 para una muestra de 83 pacientes con edades entre 1 a 14 años ^28^. En una población venezolana de 6 a 12 años de edad con y sin presencia de síndrome metabólico, se encontró un índice Tg/c-HDL de 4,43 ± 1,07 en quienes presentaban SM (n=21), y de 2,97 ± 1,65 en aquellos sin SM ^24^. Además, la media encontrada fue más elevada que la reportada en otros estudios, como el realizado en 943 alumnos de 11 a 14 años, donde se encontró una media de 1,25 ± 0,43 ^23^ o lo reportado en un estudio con población adolescente coreana que presenta un valor global de 1,74 ± 1,22 ^29^, y fue similar a lo reportado en un estudio realizado con adolescentes de la región Cajamarca en Perú, donde encontraron valores en el percentil 25, 50 y 75 de 1,62; 2,3 y 3,51, respectivamente, y asociación positiva con el IMC, colesterol no HDL y el perímetro de cintura ^30^.

Al analizar el índice de Tg/c-HDL en función al estado nutricional se encontró que quienes estaban en riesgo de bajo peso, sobrepeso y obesidad tenían un índice superior al normal, resultados similares a una población pediátrica venezolana que al segmentarla de acuerdo con el estado nutricional como normal, sobrepeso y obesidad hallaron un índice de 2,92 ± 1,24; 3,43 ± 2,2 y 3,84 ± 1,34, respectivamente ^24^.

Estas diferencias pueden deberse al tipo de población estudiada, las cuales difieren en el perfil socioeconómico, características clínicas antropométricas, hábitos de alimentación y, sobre todo, por los cambios fisiológicos y metabólicos que se desarrollan en la adolescencia, y que condicionarán ciertos rasgos en el estado nutricional como el cambio de peso corporal, alteraciones en el perfil lipídico, el aumento en la resistencia a la insulina, y la disminución en la sensibilidad de los tejidos periféricos y aumento de su secreción, dando consigo concentraciones elevadas de insulina sanguínea, y mostrando concentraciones séricas, en ayunas, de hasta dos a tres veces su valor, contrastada con lo registrado en la niñez. Basado en ello, valorar la resistencia a la insulina, ya sea midiendo directamente la insulina o haciéndolo con algún índice como el HOMA (*homeostatic model assessment*), hace que se torne difícil e impreciso durante la etapa de la pubertad o adolescencia ^23^^,^^24^^,^^31^^,^^32^.

Es notorio que dentro de nuestros hallazgos los niveles de triglicéridos, c-HDL y el IMC resultaron mayores en mujeres; mientras que los valores de los percentiles 25, 50, 75 y 95 para el índice de Tg/c-HDL fueron ligeramente mayores en varones. Esta tendencia diferenciada por sexos, ya sea total o parcial, tanto en niños como en adolescentes, se ha evidenciado en otras regiones del mundo como en Argentina, Brasil, Corea y Venezuela ^23^^,^^24^^,^^29^^,^^33^.

Un estudio brasileño evaluó a 9538 adolescentes con sobrepeso y obesidad, entre el 2013 y 2014, buscaba «Asociar el consumo de grasas trans y saturadas con dislipidemia en adolescentes», no encontró asociación significativa entre el consumo de grasas trans frente al perfil lipídico; sin embargo, sí se encontró una asociación entre el consumo de grasa saturada con el nivel de c-HDL, como lo observado en la región de Teresina, donde los adolescentes que consumían menos del 10% en grasas saturadas tenían más de dos veces la probabilidad de tener un c-HDL dentro de valores óptimos, frente a aquellos adolescente que consumían por encima del 10% en grasas saturadas ^33^.

La relación de Tg/c-HDL ha evidenciado ser un buen predictor de RI, diabetes *mellitus*, detector de partículas aterogénicas, desarrollo de enfermedades coronarias y muy ligado al desarrollo de enfermedades cardiovasculares ^29^^,^^34^^-^^37^, hipertensión arterial, enfermedad coronaria; mayor incidencia de procedimientos invasivos coronarios ^38^ y engrosamiento de la capa íntima media carotidea, considerado como un factor de riesgo de enfermedades cardiovasculares en adultos ^39^. Un estudio estadounidense determinó que el índice de Tg/c-HDL es un buen predictor de resistencia y/o rigidez de la pared arterial en adolescentes y adultos jóvenes ^40^. En otro estudio desarrollado en Italia encontraron que los niños y adolescentes con exceso de peso y con un índice de Tg/c-HDL superior a 2,2 presentaban un alto riesgo cardiometabólico con signos clínicos de daño celular y presencia de placas aterogénicas ^20^^,^^41^^,^^42^.

Frente al uso del índice de Tg/c-HDL como predicción de resistencia a la insulina, un estudio estadounidense que evaluó a 1452 jóvenes obesos, los niños y niñas de raza blanca y con un ratio mayor a 2,27, presentaron un alto riesgo de resistencia a insulina en comparación a los hispanos y afroamericanos en los que su asociación no fue estadísticamente significativa ^43^. Sin embargo, en otros estudios en población estadounidense dan como punto de corte a un índice mayor de 2,7 ^40^ y 3,5 ^38^ como indicador de enfermedades cardiovasculares existiendo hasta la fecha aún controversia sobre el punto de corte real para valorar el riesgo a desarrollar enfermedades cardiovasculares.

Si bien los puntos de corte aún son imprecisos, cabe mencionar que la media del índice encontrado en nuestro estudio resulta estar dentro del rango de 2,2 a 2,7, pero los percentiles 75 y 95 ya son cercanos a punto de corte máximo de 3,5, que fueron los índices asociados en los diferentes estudios a desarrollo de enfermedades cardiometabólicas.

Las limitaciones para este estudio fueron su diseño retrospectivo, transversal; la falta de pruebas adicionales para medir presencia o no de enfermedad cardiovascular y otras variables que pudieron afectar el resultado, como el estado socioeconómico, horas de sueño, nivel de actividad física, etc. ^42^. Se necesitaría más estudios para evaluar las asociaciones con el desarrollo de enfermedades cardiovasculares y la fluctuación de las tendencias frente a la ejecución de diferentes estrategias que estén orientadas a mejorar el perfil lipídico y nutricional de los adolescentes.

## Conclusiones

Se concluye que existe una relación positiva y estadísticamente significativa entre el índice de masa corporal con el índice de Tg/c-HDL en adolescentes atendidos en un hospital público. El índice de Tg/c-HDL fue mayor en los adolescentes con obesidad y sobrepeso superando al valor de la media poblacional. El índice de Tg/c-HDL resulta ser una herramienta práctica y económica para evaluar e identificar el riesgo de desarrollar obesidad, dislipidemia, problemas cardiovasculares, resistencia a la insulina y síndrome metabólico. 
